# Factors influencing COVID-19 testing among Native Americans and Latinos in two rural agricultural communities: a qualitative study

**DOI:** 10.3389/fpubh.2023.1220052

**Published:** 2023-09-14

**Authors:** Dillon Van Rensburg, Alexandra K. Adams, Georgina Perez, Sonia Bishop, Teresa Warne, Laurie Hassell, Thomas Quigley, Lorenzo Garza, Virgil Dupuis, Paul K. Drain, Anna Whiting Sorrell, Linda K. Ko

**Affiliations:** ^1^Institute of Translational Health Sciences, University of Washington, Seattle, WA, United States; ^2^Center for American Indian and Rural Health Equity, Montana State University, Bozeman, MT, United States; ^3^Health Systems and Population Health, University of Washington, Seattle, WA, United States; ^4^Collaborative Data Services, Fred Hutchinson Cancer Center, Seattle, WA, United States; ^5^Family and Community Engagement, Sunnyside School District, Sunnyside, WA, United States; ^6^Extension Office, Salish Kootenai College, Pablo, MT, United States; ^7^Department of Global Health, University of Washington, Seattle, WA, United States

**Keywords:** COVID-19, testing, Native Americans, Latinos, interviews, focus groups, contextual factors

## Abstract

**Objective:**

To examine factors influencing decisions to test for COVID-19 among Native Americans on the Flathead Reservation in Montana and the Latino community in the Yakima Valley of Washington state.

**Methods:**

We conducted 30 key informant interviews with community leaders and six focus groups with community members to examine factors impacting decisions to test for COVID-19 during the second year of the COVID-19 pandemic from May 2021 to June 2021.

**Results:**

Three major themes that impacted testing for COVID-19 were identified: (1) Social factors, including the influence of families and friends and employment practices; (2) health factors, including testing procedures, home-based testing, and health communication; and (3) contextual factors, including distrust for government and medical communities and the impact on cultural practices and celebrations.

**Conclusions:**

Social, health, and contextual factors influence the decision to test for COVID-19. Understanding the community's perception is critical for successful implementation of preventive strategies.

## Introduction

COVID-19 is an infectious disease that disproportionately affects racial and ethnic minority groups (1). Native Americans and Latinos report 2.8 times higher rates of COVID-19 infection as well as 5.3 and 4.6 times higher COVID-19 hospitalization rates, respectively, compared to non-Latino White individuals (2, 3). In addition, Native Americans and Latinos are estimated to have a 2.77 and 1.81 times higher likelihood of COVID-19-related deaths, respectively, compared to non-Latino White individuals even after standardizing age and place, underscoring the burden of this disease in these communities (4).

Rural agricultural communities have been heavily affected during the COVID-19 pandemic as many individuals are employed as essential workers in agricultural and food packing industries (5, 6). The influx of seasonal contracted migrant farmworkers from distant geographic areas may also place residents at risk of infection introduced by workers (7). Compounding these risks, rural agricultural communities face significant disparities in healthcare access due to a lack of healthcare infrastructure and being un- or underinsured (8, 9). Lastly, during the COVID−19 pandemic, in many areas there was an absence of coordinated emergency preparedness, inadequate local distribution of personal protective equipment, and insufficient access to tests and test results. These issues were magnified in rural communities with higher proportions of farmworkers and racial ethnic minorities (6, 7, 10).

COVID-19 testing is a critical preventive tool as it helps identify positive cases early and accurately for people to then self-quarantine, receive medical assistance, and avoid unnecessary loss of workdays (11, 12). Since the early days of the pandemic, multiple innovations were made to improve the user experience through sampling (e.g., less invasive anterior nasal swabs), collection (e.g., self-collection), and turnaround time for results (e.g., from 24–48 h to 15 min). Despite this, many individuals remained hesitant to test for COVID-19 due to a lack of trust in the government and healthcare systems, exacerbated by politicization of the pandemic and misinformation (13, 14). Given the rapid innovation and development of new tests and adjustments to a “new normal” where individuals co-exist with COVID-19, it is important to understand factors that impact acceptance of COVID-19 testing among racial and ethnic minorities living and working in rural agricultural communities.

Theoretical frameworks can help inform how people make health decisions around COVID-19 testing uptake. Social Cognitive Theory posits that individual experiences, actions of others, and environmental factors influence an individual's health behavior(s) (15). The Socio-Context Framework proposes that contextual forces (i.e., social, cultural, and religious) can directly or indirectly shape an individual's behavior (16, 17). Given the complexity of the COVID-19 behaviors, an integration of both frameworks may help capture multi-faceted constructs (i.e., psychological, behavioral, and social) that contribute to COVID-19 testing uptake. The purpose of this study is to examine factors influencing decisions to test for COVID-19 among Native Americans on the Flathead Reservation (FR) in Montana and Latinos in the Yakima Valley (YV) of Washington state, two rural agricultural communities connected by seasonal migrant workers.

## Methods

### Design overview

We conducted key informant interviews with community leaders (e.g., clinic and public health officials, and elders) and focus groups with community members to understand perceptions of COVID-19 testing and factors that influence decisions to test among Native Americans on the FR and Latinos in the YV in Washington state. This study was part of a larger community–academic partnership study, Protecting Our Communities, which promoted at-home COVID-19 testing in these rural communities (18). We had two Community Advisory Boards (CABs) advising the larger study, one from YV and another from FR. Detailed descriptions of the setting and CABs have been published (18).

The research included community representatives as co-investigators, advisors, field managers, and community health workers (CHWs). Data were collected from May 2021 to June 2021. The Montana State University, University of Washington, and Salish Kootenai College Institutional Review Boards approved this study. Participants from the key informant interviews and focus groups received a $25 incentive.

Race and ethnicity were collected to identify whether the participants were reflective of the populations of interest. On the survey, participants could select one or multiple races; ethnicity was a single option. Race included American Indian or Alaska Native, Hispanic or Latino, Black or African American, White, Asian or Pacific Islander, other (specify), and prefer not to answer. For ethnicity, the question was, “Are you Hispanic, Latino, or of Spanish origin?” Investigators developed the survey with review and direction from the Community Advisory Boards (CAB), discussed below.

### Setting and community engagement

The FR of the Confederated Salish and Kootenai Tribes is primarily located in Lake and Sanders counties of Montana with some areas extending to Missoula and Flathead counties. The FR is a rural agricultural area with more than 2,000 farms. The Lower YV includes many small agricultural communities and has a population of approximately 100,000. Roughly 67% of the population is Latino with 95% being Mexican American (19). During the growing season, migrant workers from YV frequently travel to the Flathead Valley for harvesting. This is a unique connection between the communities.

We had two CABs advising the project. The CAB for the FR consisted of tribal leaders, health professionals at tribal clinics and private hospitals, and public health professionals. The YV CAB was composed of representatives from community organizations, local health departments, community health centers, and schools. The CABs met bimonthly. Additionally, the investigators' team consisted of two community co-investigators representing the FR and YV. FR co-investigator was a member of the Confederated Salish and Kootenai Tribes and the YV co-investigator was an active community advocate and respected member of the Latino community in the YV.

### Participants

#### Key informant interviews

Purposive sampling was used to recruit key informants. The research team collated contact information from local community leaders (e.g., incident commanders, program directors), augmented by CAB members' suggestions. Trained CHWs recruited, screened, and consented community leaders for an interview. CHWs in the YV were bilingual and bicultural. Interviews took place via a HIPAA-approved virtual platform. Thirty key informant interviews (i.e., 15 FR, 15 YV) were conducted. Interviews lasted 40–60 min, were audio-recorded, and transcribed. Table 1 shows interviewees' demographic information.

**Table 1:** Demographics of the interview and focus group participants.

	**Yakima interviews (*N =* 15)**	**Yakima focus groups (*N =* 19)**	**Flathead interviews (*N =* 15)**	**Flathead^a^ focus groups (*N =* 20)**
**Gender (** ***n*** **,%)**				
Male	1 (7)	3 (16)	4 (27)	9 (45)
Female	14 (93)	16 (84)	11 (73)	11 (55)
**Race (** ***n*** **,%)** ^b^				
Black/AA^c^	0 (0)	0 (0)	0 (0)	1 (5)
AI/AN^d^	0 (0)	0 (0)	13 (68)	16 (64)
Latino	11 (73)	17 (90)	2 (11)	1 (4)
White	4 (27)	0 (0)	4 (21)	7 (28)
Other	0 (0)	1 (5)	0 (0)	0 (0)
**Language (** ***n*** **,%)** ^e^ ^,^ ^f^				
Bilingual (English dominant)	6 (40)	8 (42)	0 (0)	0 (0)
Bilingual (Spanish dominant)	5 (33)	3 (16)	0 (0)	0 (0)
Bilingual (Native and English)	0 (0)	0 (0)	2 (14)	16 (80)
English only	0 (0)	0 (0)	11 (73)	3 (15)
Spanish only	0 (0)	8 (42)	0 (0)	0 (0)
Prefer not to answer	4 (27)	0 (0)	0 (0)	0 (0)
**Education (** ***n*** **,%)**				
Elementary school	0 (0)	3 (16)	0 (0)	0 (0)
Some high school	1 (7)	4 (21)	0 (0)	0 (0)
High school graduate/GED	1 (7)	7 (37)	0 (0)	0 (0)
Technical school diploma	0 (0)	1 (5)	0 (0)	0 (0)
Some college	4 (27)	2 (10.5)	4 (27)	6 (30)
College graduate	6 (40)	2 (10.5)	6 (40)	13 (65)
Graduate school degree	3 (20)	0 (0)	5 (33)	1 (5)
**Birth country (** ***n*** **,%)**				
United States	8 (53)	6 (32)	15 (100)	20 (100)
Outside of the United States	7 (47)	13 (68)	0 (0)	0 (0)
**Employment (** ***n*** **,%)**				
Full time	12 (80)	10 (53)	14 (93)	15 (75)
Part time	2 (13)	1 (5)	1 (7)	0 (0)
Unemployment	0 (0)	3 (16)	0 (0)	2 (10)
Other	1 (7)	4 (21)	0 (0)	3 (15)
Prefer not to answer	0 (0)	1 (5)	0 (0)	0 (0)
**Annual household income (** ***n*** **,%)**				
Less than $15,000	0 (0)	2 (10.5)	0 (0)	0 (0)
$15,000–less than $35,000	2 (13)	4 (21)	5 (33)	2 (10)
$35,000–less than $50,000	5 (33)	8 (42)	0 (0)	10 (50)
$50,000–less than $75,000	3 (20)	3 (16)	3 (20)	3 (15)
$75,000 or more	4 (27)	2 (10.5)	5 (33)	3 (15)
Don't know/prefer not to answer	1 (7)	0 (0)	2 (14)	2 (10)

a 21 interviews were transcribed from the Flathead Reservation focus groups but only 20 participants completed the demographic survey.

b One participant did not respond to the question about race from the Yakima Valley focus groups.

c AA = African American.

d AI/AN = American Indian and Alaskan Native.

e Two participants from the Flathead Reservation key informant interviews did not respond to the question about language.

f One participant from the Flathead Reservation focus groups did not respond to the question about language.

#### Community focus groups

Purposive sampling was also used to recruit focus group participants. Participants comprised community members who lived and/or worked in one of the two rural agricultural communities. Previous study participants from the “Together We STRIDE” (20) and “Healthy and Sustainable Diet for All” (21) studies who agreed to be contacted for future studies were called for screening and consenting. When 10 participants were enrolled, the CHWs scheduled a date and time for the focus groups. A total of six focus groups were convened (i.e., three from the FR, three from the YV). Two focus groups in the YV were conducted in Spanish.

On the FR, 26 community members were contacted via phone and enrolled. Of the 26 enrolled, 21 participated in one of three focus groups; none of the participants from the FR refused to participate in the study but five were no-shows. In the YV, 55 community members were contacted via phone and 19 were enrolled; 16 refused to participate (i.e., not interested, not enough time for them to participate, no access to a phone or computer for focus groups), 15 were unable to be contacted, and five were no-shows. YV participants completed one of three focus groups: English-language (*n* = 8), Spanish-language (*n* = 5), and Spanish-language (*n* = 6). Sessions were facilitated by an experienced bilingual/bicultural moderator. Written informed consent was administered in English or Spanish prior to the discussion. All focus groups lasted 45–60 min, were audio-recorded, and transcribed. Table 1 shows focus group participants' demographic information.

#### Instrumentation

The interview and focus group guides were designed to understand perceptions and factors influencing COVID-19 testing. Social Cognitive Theory and Socio-Context Framework informed development and analysis. Examples of interview and focus group questions in line with the Social Cognitive Theory were, “What do you think will motivate people to use the home-based testing?” and “What is the role of family/friends in people's decision to test for COVID-19?” Examples of interview and focus group questions grounded in the Socio-Context Framework were, “Can you tell us any beliefs in your community that can make it easy or difficult for people to get tested for COVID-19” and “Can you tell us any experiences in your community where people's financial situation became a barrier for getting tested for COVID-19?”

### Data analysis

Two research team members used the interview guide to create a tentative codebook to analyze key informant interview data. The codebook was used by three data analysts to conduct deductive coding to capture preliminary constructs. Using an iterative process, the three analysts met weekly to refine the codebook, adding, removing, and revising codes as needed to address inter-rater agreement and compare new data with existing data. To capture discussions that emerged beyond the a priori questions, we used an inductive, constant comparison approach to identify themes (22). Throughout the coding and analysis process, the analysts built consensus around the themes, compared themes arising from the data, and determined possible linkages across participants and thematic categories. Data were coded using Dedoose version 9.0.62. Themes were then validated with the CAB and community co-investigators. The research team used the interview codebook as a preliminary codebook for the focus group analysis. The focus group codebook was used by two data analysts, following the coding procedure described above.

## Results

A total of 69 key informant interviewees (15 FR, 15 YV) and focus group participants (20 FR, 19 YV) participated in the study. In the FR, the majority of participants in the interviews were female (73%), while the focus group participants were split evenly (55% female, 45% male). More than half of participants self-reported being American Indian (68% interviews and 64% focus groups). Many participants in the interviews reported being English dominant (73%) while the focus group participants reported being bilingual in their Native and English language (80%). In the YV, the majority of participants in the interviews and focus groups reported being female (93% interviews and 84% focus groups), Latino (73% interviews and 90% focus groups) and bilingual with slightly more participants reporting English dominant.

We identified three themes and eight subthemes for factors that impact decisions to test for COVID-19. The themes and subthemes include: (1) social factors (influence of families and friends, employment practices), (2) health factors (testing procedure, self-testing at home and health communication), and (3) contextual factors (distrust for the government and medical community and disruption to cultural practices and celebrations).

### Social factors

#### Influence of families and friends

Most participants mentioned their desire to protect families as a motivator to test for COVID-19. Participants noted how family members encouraged each other to get tested and follow other COVID-19 safety precautions (e.g., vaccination). An individual from the FR interviews described testing as “an opportunity to lead by example” and be a good model for their family. While participants from the YV used “family” to refer to their immediate and extended family members, FR participants referred to communities at large as part of their family, extending their sense of responsibility.

“*I think [the role of family or friends in people's decision to test for COVID-19] is a huge deal. There's been almost like peer pressure for adults... If I were coughing, I could see myself saying, “Well, I'll just stay home for a day and see how I feel.” But I have a wife who would grab me by the ear and drag me into the hospital to get tested. I know when my daughter who lives in Missoula called and said, “I'm not feeling great, Dad,” I said, “Go get tested.”*

While several participants from the FR and YV focused on “good” pressure that family and friends exert to comply with safety precautions, a few FR and YV key informant interview participants felt that was not their role—the decision not to adhere to COVID-19 guidelines is ultimately a person's choice. A few FR and YV focus group participants mentioned a social divide in some families regarding decisions to adhere to COVID-19 safety precautions.

#### Employment practices

Participants from both locations shared that mandatory quarantine periods from a positive COVID-19 test and missed wages from non-paid leave were reasons not to test. Many participants from the YV focus groups emphasized inconsistent practices on when employees return to work. Some employers allowed workers to come back after evidence of a negative test result, while others asked employees to quarantine using vacation hours.

“*…there was a lot of concern in the beginning…if I'm not feeling well, and I go get tested, then I'm off to quarantine which results in missed work and missed income. Before they had…the leave, I know a lot of people were like, ‘I'm not going to get tested and I'll just wait it out'…”*

Several participants from the FR and YV focus groups recalled situations where people could “not afford to test positive.” Considering there are no incentives to get tested for COVID-19, the potential of getting a positive result would be too financially risky. In addition, YV interview and focus group participants shared that deportation and job loss were serious concerns among farmworkers and that testing positive for COVID-19 could put them at risk.

“*A lot of people don't have sick leave and so it's hard for them to miss work and some people will lose their job just for not showing up if they work at a dairy or if they work at a warehouse or at a fast-food place. Some people could lose their jobs just for not showing up to work.”*

### Health factors

#### Testing procedure

Interview and focus group participants from both communities agreed that pain and discomfort from swabbing were barriers to COVID-19 testing. Participants talked about swabs being inserted in the back of the nasal cavity. While there was a consensus that test results were clear and easy to understand, long wait times at testing centers was commonly mentioned as anxiety-inducing. Inconsistent wait times for test results was also mentioned by several focus group participants from the FR; some received results within a few days while others waited over two weeks. Participants also shared fears that results may be fabricated, and that infection is introduced by intentional use of infected nasal swabs.

“*It's uncomfortable. The test sucks'cause they don't do – [the swab] is not little – it's the one that…goes deeper in your nostril… It's an uncomfortable test. You don't want to get it if you don't have symptoms.”*

#### Home-based testing

Convenience and privacy were the most reported motivators for at-home testing among the FR and YV interview and focus group participants. Some individuals valued the ability to complete the test in the privacy of their home. Others valued the privacy of results (i.e., reduced the chance of others knowing the result). A few individuals from the FR and YV stated at-home testing also allows them to circumvent COVID-19 exposure from waiting in line at a community testing site. YV focus group participants also mentioned benefits for those who do not have vehicle access.

“*Not feeling like their privacy is being invaded and not having to go to the doctor's if they have a fear of the doctor's office in the first place'cause I have a lot of clients with anxiety and they do not want to go to the doctors. So, removing that from the equation might help.”*

However, concerns were also raised about commercially available home-based tests sent to a laboratory for test results. YV focus group participants raised concerns for privacy and information safety, such as fear that nasal samples will be kept by institutions or private companies for future use without their consent. Some participants also mentioned concerns around the legitimacy of test results, mentioning that at-home tests do not provide the same level of accuracy as tests given by medical professionals. Others noted that at-home tests could give inaccurate results because swabbing is done by a lay person and not a trained medical professional. Several participants from both locations noted that having clear and simple instructions on how to use self-tests is important for at-home testing. The need for detailed instructions identifying test drop-off locations was noted. Several YV interview participants also discussed how healthcare portals and apps were inconvenient due to the time and difficulty of setting up an account.

#### Health communication

Many FR and YV interview and focus group participants described COVID-19 testing as “lifesaving” by identifying cases early and leading individuals to receive rapid treatment. They also shared that this information needed to be emphasized to motivate people to take the test.

Participants from both communities requested access to more COVID-19 resources. Participants from the YV wanted education for employers on instituting workplace policies for employees, particularly migrant farmworkers, following exposure to COVID-19. Interview participants also stressed that employers across different organizations needed to be “on the same page” and follow COVID-19 procedures consistently. Interview participants also mentioned needs for access to information about evolving guidelines and testing eligibility, as testing was thought to be less beneficial among those fully vaccinated for COVID-19. A few focus group participants said the information should be delivered by trusted community members to increase testing uptake.

“*I think [we need] to make sure that all employers have the same information, I think it should be the same policy for everyone if you get exposed or you get COVID because some employers say, ‘Oh, that's fine. If it's negative, you can come back to work' but some of the employers said, ‘No, even though you're negative, you still have to say home and use your vacation time.' So, I think it would be nice if all the employers kind of have the same policy.”*

Both communities discussed social media as a source for COVID-19 information and an influence on the decision to test. Several interview participants from both communities expressed concerns about the credibility of the information, as most social media posts about COVID-19 safety protocols (e.g., testing, vaccination) were widely negative (e.g., deaths attributable to doctor's malpractice, government tracking) and not representative of the average person's experience. Some focus group participants from the FR preferred social media over other media (e.g., television) for receiving information and as a strategy to dispel misinformation regarding COVID-19 testing.

### Contextual factors

#### Distrust for the government and medical community

Interview and focus group participants mentioned that distrust for the government and medical community percolated into individuals' testing decisions. Several FR participants noted government actions are often met with skepticism because of injustices done to Native peoples, such as introduction of infectious diseases by non-Indigenous settlers, scientific malpractice, and low quality of care for tribal communities. While there were limited discussions among the YV participants regarding distrust for government and medical community, a YV interviewee explicitly shared that some Latinos share a culture of distrust for the medical system because of historical marginalization.

“*I think some communities are just naturally a little distrustful of the medical community. I know that I heard African American communities are kind of similar because of the same kind of historical events that have happened there. I also think that doctors and nurses can sometimes be pretty condescending. And I know I've had that experience. So that kind of makes it even more that way where we don't trust because we've been treated kind of bad, like we don't know things.”*

#### Disruption to cultural practices and celebrations

As this study took place during the early stages of the pandemic, several FR and YV interview and focus participants raised disappointment in not being able to participate in social, cultural, and spiritual events because of COVID-related gathering restrictions. Gathering together for family events and pow wows, or wintertime dances, and harvesting of culturally significant foods were noted as deeply rooted in family and community culture in both locations. However, people understood the need to cancel such events and applauded community response toward the gravity of the pandemic. Participants also saw COVID-19 testing and other preventive measures (e.g., vaccination) as ways to lower infection rates and reopen celebrations to family and community members again and encouraged use of this messaging for testing promotion.

“*There was a while where we weren't seeing anyone, and that was difficult for our entire family. Like I said before, our whole culture is based on relationships. So, it was a struggle for quite a while. We're starting to slowly gather now, but most of our family has been vaccinated and we're trying to still be cautious. You can see how much people have missed those gatherings and taking chances to do certain things like wintertime dances and stuff.”*

## Discussion

This study examined how decisions to test for COVID-19 are shaped among Native Americans and Latinos from two rural agricultural communities, the FR in Montana and the YV in Washington state. Decisions to test are shaped by social, health, and contextual factors. For social factors, our study found that social tightness and collective responsibility for the children and older generations influenced the decision to test among Native Americans and Latinos. The concept of family, with inclusion of extended family members, is a cornerstone of most Native American communities. It extends across the generations as children are viewed as the future of Tribes who will preserve Tribal beliefs and traditions and elders are the wisdom keepers of the Tribal culture (23). The tight social network and exchange of support in times of need among Latinos have also been extensively described in the literature (24). Our study corroborates these findings and suggests that the strong sense of responsibility among Native American and Latinos to protect their families and communities is a key motivator to test for COVID-19. This sense of honoring their family and community's health was used to motivate others to test, either through exerting positive pressure on others or through modeling without patronizing individuals' rights to make their own decisions.

Health and contextual factors, especially around mistrust for medical community and the government, co-constituted participants' decision to get tested for COVID-19. Our study participants had a number of concerns about testing procedures, including swabbing, receiving results, and data privacy. While multiple innovations have improved the user's experience with sample collection, rapid return of results, and accuracy of results, there may be lingering concerns about data privacy in the community. Pervasive mistrust for medical community and the government has been reported given the magnitude of past misconduct and exploitation of Native Americans and Latinos by these organizations (25). Concerns around storage of nasal samples and their further use without prior consent are examples of participants' reflections on past experiences that influence their current health decisions. When FR participants were asked about community beliefs, there was a dynamic discussion about historical trauma and injustices done to Native peoples by non-Natives. Similar distrust for the medical system due to historical marginalization was also mentioned by Latino participants. Rather than approaching the Native American and Latino communities from the outside when promoting testing behaviors, public health agencies should find trusted messengers in the respective communities and provide them with resources to enhance community modeling across all COVID-19 preventive behaviors.

Discussion around health factors extended to the benefits of having access to accurate health communication. Health communication was seen as a tool to address many issues in the community, including educating employers. The issues employees faced during the pandemic were in large part due to workplace policies, such as missed wages from non-paid quarantine time, lack of sick leave, and in YV the potential risk of deportation due to lost work time. Participants shared that education around evolving challenges, policies, and guidelines of the COVID-19 pandemic may be a solution to protect employees. Rather than expecting that health communication will be the solution, state and federal governments should institute stronger policies to protect essential workers in agricultural communities and use communication to educate employers on deploying the policies.

Our study participants also discussed health communication as a tool to correct misinformation and culturally align COVID-19 messages with community values. Participants mentioned an overabundance of misinformation around COVID-19 testing and vaccination in social media. Multiple studies demonstrated that the role of digital platforms is as much a threat to public health as the virus itself during the COVID-19 pandemic (26). While our study participants acknowledge the negative information on social media, they also saw the benefits of dispelling and correcting misinformation through social media. Public health agencies should collaborate with local community leaders and trusted messengers and build a larger partnership with social media companies to magnify credible information. Local community leaders/local social influencers should be trained to evaluate social media posts and resources, and social media companies should adapt algorithms to prioritize credible health information in search indices (27). Additionally, public health agencies should align their messages with community values, such as highlighting the benefit of testing to accelerate a safe return to cultural events.

Consistent with the Social Cognitive Theory, our study participants reported how their social environment influenced their decision to get tested for COVID-19. Participants described a commitment to protect family and friends as a source of motivation to get tested. Some participants emphasized the role of observational learning in shaping their health behaviors. By observing family members and friends who engaged in COVID-19 preventative behaviors, such as getting tested, some participants were more inclined to adopt similar practices themselves. This highlights the significance of modeling and vicarious reinforcement within the theory. Similarly, with Socio-Contextual Framework, participants detailed contextual factors directly or indirectly shaping their behavior related to COVID-19 testing. Participants cited distrust in the government and medical communities as a factor affecting their engagement in COVID-19 testing. This distrust stems from historical experiences of marginalization and scientific malpractice inflicted upon their communities (28, 29). These contextual factors highlight the significance of broader social, historical, and systemic influences and how these factors can shape individual behavior and decision-making processes when it comes to COVID-19 testing and beyond.

This was the first study to examine factors influencing decisions to test for COVID-19 among Native Americans on the FR and Latinos in the YV of Washington state, two rural agricultural communities connected by seasonal migrant workers. One limitation of this study is that participants were from rural agricultural communities, which were most affected during the pandemic. Native Americans and Latinos from urban communities may have different experiences. Another limitation is that participants in this study were mostly female (*n* = 52, 76%) and, due to the distribution of our cohort by sex and the nature of qualitative research studies focusing on narratives, the study team could not compare experiences between men and women regarding COVID-19 testing uptake. The study was also conducted in 2021 at the height of the COVID-19 pandemic. While COVID-19 testing is not a high priority at the present moment, testing is a new normal, therefore, understanding factors that shape decision making for COVID-19 testing is relevant, especially in communities most affected by the pandemic.

Understanding the community's perceptions and experiences around COVID-19 testing is critical for successful implementation of strategies to increase testing uptake. Our study highlights unique social, health, and contextual factors that shape decisions to test for COVID-19 among Native Americans and Latinos from rural communities. Strategies for testing uptake among these communities need to be grounded in partnerships with the local community to leverage credible, trusted, influential community champions; state government to develop and institute workplace policies that safeguard employees; and social media companies to adapt algorithms to prioritize credible messages.

## Data availability statement

The original contributions presented in the study are included in the article/supplementary material, further inquiries can be directed to the corresponding author.

## Ethics statement

The studies involving humans were approved by the Institutional Review Boards from Montana State University, University of Washington and Salish Kootenai College. The studies were conducted in accordance with the local legislation and institutional requirements. The participants provided their written informed consent to participate in this study.

## Author contributions

DV and GP contributed to the data analysis, interpretation of the data, manuscript development, and revision. AA, LG, VD, and PD contributed to the conceptualization and the design of the study, data interpretation, and revision of the manuscript. SB contributed to the implementation of the study, data collection, data interpretation, and revision of the manuscript. TW and LH contributed to the implementation of the study, data interpretation, and revision of the manuscript. TQ contributed to the data analysis, data interpretation, manuscript development, and revision of the manuscript. LK contributed to the conceptualization and the design of the study, data analysis data interpretation, manuscript development, and revision. AW contributed to data interpretation and revision of the manuscript. All authors contributed to critical revision of the manuscript for important intellectual content and have approved the current version.
